# Effects of subchronic inhalation exposure to an organophosphorus insecticide compound containing dichlorvos on wistar rats’ otoacoustic emissions

**DOI:** 10.1016/j.bjorl.2020.04.005

**Published:** 2020-05-19

**Authors:** Aléxia dos Reis, Eduarda Oliveira Cunha, Marina Tuerlinckx Costa Valle, Márcia Salgado Machado, Eliane Dallegrave

**Affiliations:** a Universidade Federal de Ciências da Saúde de Porto Alegre, Laboratório de Pesquisa Toxicológica, Programa de Pós-Graduação em Patologia, Porto Alegre, RS, Brazil; b Universidade Federal de Ciências da Saúde de Porto Alegre, Laboratório de Pesquisa Toxicológica, Programa de Pós-Graduação em Ciências da Saúde, Porto Alegre, RS, Brazil; c Universidade Federal de Ciências da Saúde de Porto Alegre, Laboratório de Pesquisa Toxicológica, Departamento de Fonoaudiologia, Porto Alegre, RS, Brazil; d Universidade Federal de Ciências da Saúde de Porto Alegre, Laboratório de Pesquisa Toxicológica, Programa de Pós-Graduação em Patologia, Departamento de Fármaco-Ciências, Porto Alegre, RS, Brazil

**Keywords:** Pesticides, Audiology, Rats, Toxicology

## Abstract

**Introduction:**

Considering that previous studies suggest that pesticides may cause hearing disorders in humans, as well as the lack of studies proving the specific mechanisms of injury and the difficulty of separating concomitant etiological factors of the hearing damage, such as noise and vibration, it is important to develop studies using animal models to elucidate the effects of exposure to those substances isolated from other hearing damage etiologies.

**Objective:**

To evaluate if the exposure to a dichlorvos based organophosphorus insecticide may induce ototoxicity.

**Methods:**

36 male Wistar rats were assigned to 3 groups (12 rats/group): control (exposed to water), positive control (treated with cisplatin to induce hearing damage) and experimental (exposed to dichlorvos based organophosphorus insecticide). The amplitude of distortion product otoacoustic emissions in the frequencies of 4, 6, 8, 10 and 12 kHz was evaluated before and after exposure, as well as systemic toxicity signs, body mass gain and plasma cholinesterase. Open field and plus maze tests were performed in 24 rats: experimental (*n* = 8), control (*n* = 8) and positive control group (*n* = 8 introduced new rats to induce anxiolytic activity) to evaluate the locomotor activity and anxiety, respectively.

**Results:**

There was no significant change in body mass gain and plasma cholinesterase in the dichlorvos based organophosphorus insecticide group, however, the animals showed transient piloerection, depression and dyspnea during exposure. The behavior was not affected in any group. The frequencies of 8 and 10 kHz were significantly affected bilaterally in the insecticide group, which also showed a significant difference of the control in 10 kHz on the right and 8 and 10 kHz on the left ear.

**Conclusion:**

Subchronic inhalation exposure to dichlorvos based organophosphorus insecticide induced ototoxicity in the cochlear function of rats without relevant systemic toxicity.

## Introduction

According to the literature, exposure to pesticides may cause alterations in human hearing.^1^, ^2^, ^3^, ^4^, ^5^, ^6^ Auditory injury is often due to several factors^7^ and, when it occurs occupationally in the primary sector of the economy, it may be related primarily to two factors: exposure to ototoxic chemicals, such as solvents and pesticides, as well as noise from agricultural machinery. The hearing loss due to ototoxic drugs’ exposure is similar to the one that occurs at high sound pressure levels, being sensory-neural, bilateral, symmetrical, irreversible, at high frequencies and damaging to the hair cells of the cochlea.^8^

Agrochemicals belonging to the class of Organophosphates (OPs) are widely used in agriculture as insecticides. Among them is dichlorvos (2,2 Dichlorovinyl Dimethyl Phosphate – DDVP), used in public health campaigns to control vectors, as a resource in veterinary medicine – in the fight against ectoparasites^9^ in the primary sector of the Brazilian economy (agriculture). OPs act as cholinesterase inhibitors and they are responsible for many intentional or accidental poisonings.^10^

OPs are important to control vectors considering that Brazil has a high number of reported cases of dengue, chikungunya and Zika, and nowadays the country could be considered as the most suffering one from *Aedes aegypti*.^11^ Therefore, insecticides, such as organophosphates, are widely used in public health for vector control. Dichlorvos, an OP, is also used in domestic disinfestations. Furthermore, there is a concern about its indoor use that may be related to critical phases of human development. Children can be exposed to pesticides, considering that they tend to play and explore their environment, touching surfaces as the floor^12^ and have a hand-to-mouth behavior, absorbing more pesticides than adults.^13^

Due to the existence of several etiologic agents that cause occupational hearing loss, such as machinery noise and exposure to chemical agents, it is difficult to correlate this auditory pathology. Furthermore, even those who do not work directly with these substances are exposed to them and hearing loss may be multifactorial.^7^

The study in animals exposed only to pesticides, free from the bias such as noise and vibration, allows a characterization of the hearing loss and the study of its mechanisms. In the literature, there are few studies evaluating ototoxicity due to exposure to agrochemicals in animal models, and these often use routes not correlated with occupational exposure, such as intraperitoneal injection.^14^, ^15^, ^16^ Therefore, the present study has aimed to evaluate the ototoxic effects based in cochlear function of the subchronic inhalation exposure of DBOI in rats through distortion product otoacoustic emissions.

## Methods

### Chemical agents

A commercially available emulsifiable concentrate formulation (55%) of organophosphorus insecticide containing dichlorvos as the active ingredient was diluted in distilled water at the concentration of 0.0015 mg/mL, corresponding to 1/10 of rat inhalation LC50, which is 15 mg/L^17^ (the LC50 refers to the concentration that causes the death of 50% of the exposed animals). The commercial product was chosen in order to use the substances to which the population is in fact exposed.

As a positive control for ototoxicity the antineoplastic drug cisplatin was chosen. Rats were treated with 8 mg/kg intraperitoneally, once daily, for 3 consecutive days. Cisplatin were diluted in physiological solution (10 mL of solution/kg).

### Experimental animals

In the present study, 36 male Wistar rats (*Rattus norvegicus*) age 60 days and weighing approximately 300 ± 50 g were used. The animals were kept under controlled conditions of the bioterium, 12 h light/dark cycle, receiving water and food ad libitum, except during exposure.

### Experimental

The experimental protocol was adapted from the subchronic inhalation toxicity test (repeated exposure), number 413, from the Organization for Economic Co-operation and Development (OECD) Guidelines for the Testing of Chemicals^18^ and approved by the Federal University of Health Sciences of Porto Alegre Ethics Committee on Animal Use Ethics Committee on Animal Use under n° 321/15 and all the procedures of health care (according to the National Council for the Control of Animal Experimentation – CONCEA) were followed in order to avoid pain or distress in animals. Only animals with no signs of external ear pathology (verified by otoscopy performed by a veterinarian) and Distortion Product Otoacoustic Emissions (DPOAE) present in all tested frequencies (4, 6, 8, 10 and 12 kHz) were selected for the experiment.

The inhalation exposure chambers were soundproof, having 56 L of volume and were coupled to ultrasonic nebulizers (avoiding noise) as an inlet stream, and to an aspirator as an exhaust system. There were 4 animals per chamber, considering the volume established by the OECD.

The sample size was based on previous studies, using the variability in the responses to the auditory parameters evaluated in rats exposed to the ototoxic agent cisplatin as a positive control.^19^

During the adaptation period (5 days) the animals were trained to habituation with the instruments of auditory evaluation, as well as adaptation to the period in the exposure chamber, initially for 1 h (day 1), the 2^nd^ day for 2 h, the third day for 3 h and on the 4^th^ day for 4 h (using only air flow). On day 5 air flow was used with water vapor for 4 h.

The rats (*n* = 36) were randomly assigned to three groups: negative control, positive control (for hearing damage – cisplatin) and experimental. The negative control group consisted of 12 rats exposed to water (vehicle for dilution of the formulation) by inhalation for 4 h, 5 times a week, for 6 weeks. The positive control group included 12 rats treated with 8 mg/kg cisplatin intraperitoneally, once daily, for 3 consecutive days (totaling 24 mg/kg). The experimental group consisted of 12 rats, exposed to inhaled DBOI at a concentration of 0.0015 mg/mL, for 4 h, 5 times a week, for 6 weeks.

### Assessments

#### Clinical signs and body mass

The animals were evaluated for body mass and clinical signs (depression or excitation, tremor, piloerection, dyspnea) throughout the experimental period (once a day on exposure days).

#### Distortion product otoacoustic emissions (DPOAE)

Distortion product otoacoustic emissions were performed in the control and experimental groups before (pre-exposure) and after (post-exposure) the exposure period (0 and 42 days), and in the positive control to induce hearing damage (cisplatin) immediately before the 1^st^ administration and 24 h after the 3^rd^ administration in the frequencies of 4, 6, 8, 10 and 12 kHz. This assessment was performed without any type of anesthetic, only with previous habituation of the animals to the instruments of auditory evaluation. DPOAE were recorded using two tones (f1 and f2) as acoustic stimuli (f1/f2, f2:f1 ratio fixed at 1.22). The f1 and f2 tones were presented at a stimulus level of 65 and 55db SPL (Sound Pressure Level). DPOAE tests were performed with an infant size hearing probe placed into the external ear canal of the rat and tested at frequencies of 4, 6, 8, 10 and 12 without use of anesthesia. Distortion product otoacoustic emissions were performed in the control and experimental groups before (pre-exposure) and after (post-exposure) the exposure period (0 and 42 days), and in the positive control previously the 1^st^ administration and 24 h after the 3^rd^ administration. Although the normality criterion for otoacoustic emissions often considered is at least 6 dB of signal/noise ratio, in this research the DPOAE were analyzed by the amplitude of response before and after exposure, which was compared between groups (control, positive control for hearing damage and DBOI). This occurred, once the terms “normal” and “altered” are more suitable for transient emissions. In this current research, DPOAE (and pre-and post-exposure response amplitude evaluation) have become a better option, once they show a reduction in response amplitude, even if, according to the 6 dB criterion, there is normality.

#### Behavior tests

30 days after the start of the experiment, eight rats from each group (control and experimental group) were randomly selected and, in addition, 8 new rats were included in a positive (anxiolytic) control group (extra rats selected apart from those pre-defined groups) treated with intraperitoneal diazepam (1 mg/kg) 30 min before the behavioral test (Plus Maze) to induce anxiolytic activity.

#### Open field test

The open-field arena is intended to quantify general spontaneous locomotor activity. For that purpose, a black arena (60 × 60 × 30 cm) divided into 16 squares was used. The animals were positioned in the posterior left corner and monitored to analyze the exploratory profile for five minutes. The following parameters were evaluated: number of displacements (when the animal places all four legs in any of the squares, quantified as external crossings the quadrants close to the borders of the box and internal quadrants positioned in the center of the apparatus; this parameter is associated with locomotor activity); latency to leave the first quadrant (associated with anxiety); number of rearings (the number of times the animal is raised on the hind legs); grooming time (time spent cleaning up). At each animal exchange the arena was cleaned.^20^, ^21^

#### Plus maze

This test is used to evaluate anxiety. The equipment used is a wooden labyrinth, cross-shaped, with a central square (12 × 12 cm) and each of the four arms measure 50 cm long by 10 cm wide. Animals were placed in the center of the maze with the head facing the closed arm. The behaviors were recorded during the 5 min of exposure and the parameters evaluated were: time of permanence in the open and closed arms, numbers of entries in open and closed arms, numbers of head dippings (when the animal makes a movement similar to a dive to observe the surface), and time spent in the center of apparatus.^22^, ^23^

### Euthanasia

At the end of the experimental protocol (24 h after the last inhalation exposure or treatment with cisplatin) euthanasia was performed using prior anesthesia with sodium thiopental administered intraperitoneally (40 mg/kg) associated with lidocaine (10 mg/mL). Under anesthesia, the rat was incised in the abdomen and blood was collected from the caudal vena cava and transferred to a tube containing EDTA, centrifuged, and the plasma placed in eppendorf and frozen until the analysis. The animals’ organs (liver, kidneys, spleen, heart and lungs) were removed and inspected for macroscopic alterations. Rat plasma was used for the determination of plasma cholinesterase activity by a kinetic enzymatic assay.

### Statistics

The DPOAE data were presented as median and interquartile ranges and the comparison between the groups was performed by Mann–Whitney (post-exposure assessment in relation to the pre-exposure of each group) and Kruskal–Wallis (comparison of the variations between groups) following Dunn's post hoc. Parametric data were presented as mean and standard error of the mean and those variables that had normal distribution were evaluated by Student's *t* test (relative body mass and cholinesterase activity). All tests considered a confidence interval of 95%. The statistical analyses were performed by the Statistics Software Package (SPSS version 25.0 for Windows, SPSS Inc., Chicago, IL, USA).

## Results

**Figure 1:**
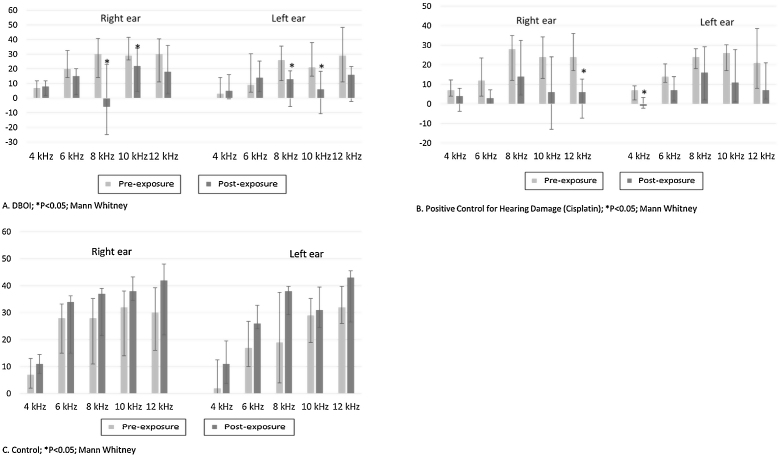
Median and interquartile ranges of DPOAE amplitudes pre- and post-exposure to DBOI, cisplatin (positive control por hearing damage) and water (control).

Exposure to the DBOI significantly reduced post-exposure DPOAE (*p* < 0.05, Mann–Whitney) compared to pre-exposure measurements (Fig. 1) at frequencies 8 and 10 kHz in both ears (*p* = 0.034 and 0.045 on the right and *p* = 0.015 and 0.013 on the left ear, respectively). As well, the cisplatin treatment significantly reduced DPOAE at (Fig. 1) frequencies 12 kHz (*p* = 0.006) in the right ear and 4 kHz in the left ear (*p* = 0.032). The control group showed no significant difference (*p* > 0.05; Mann–Whitney) in post-exposure assessments in relation to pre-exposure (Fig. 1), confirming the standardization of the experimental model.

**Figure 2:**
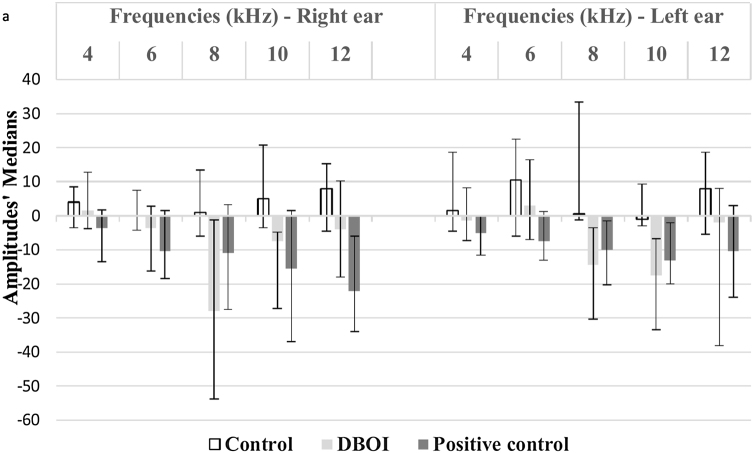
Variation of the amplitudes (medians and interquartile ranges) between the pre and post-exposure period to the control (water), positive control and DBOI.

There was a statistically significant difference between the groups (*p* < 0.05; Kruskal–Wallis) in relation to the variation of the medians post- and pre-exposure (Fig. 2).

According to the *p*-value presented in Table 1, the DBOI group showed a significant difference (*p* < 0.05) from the control at frequency 8 kHz on the right and at frequencies 8 kHz and 10 kHz in the left ear. Although at the frequency of 10 kHz on the right ear the analysis showed a difference between the groups (*p* = 0.026, Kruskal–Wallis), the comparison of means did not identify which groups differ from others. The cisplatin group showed a significant difference in relation to the control group at frequency 8 kHz on the left and in relation to DBOI group at frequency 12 kHz on the right ear (Table 1).

**Table 1:** Kruskal–Wallis and Dunn's post hoc tests.

Ear	Frequency	Kruskal–Wallis	Dunn's post hoc		
			Control × DBOI	Control × Cisplatin	Cisplatin × DBOI
Right	4 kHz	*p* = 0.109			
	6 kHz	*p* = 0.199			
	8 kHz	*p* = 0.045	*p* = 0.040		
	10 kHz	*p* = 0.026	*p* = 0.053	*p* = 0.063	*p* = 1.000
	12 kHz	*p* = 0.017			*p* = 0.014
Left	4 kHz	*p* = 0.183			
	6 kHz	*p* = 0.146			
	8 kHz	*p* = 0.004	*p* = 0.005	*p* = 0.039	
	10 kHz	*p* = 0.014	*p* = 0.013		
	12 Hz	*p* = 0.136			

**Figure 3:**
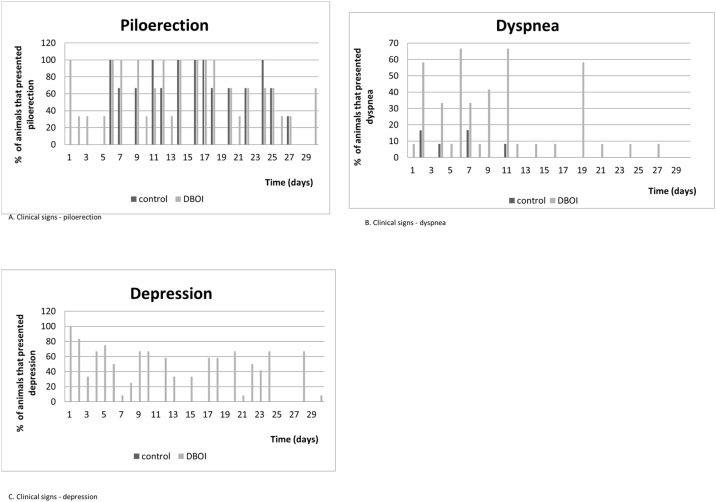
Clinical signs.

The relative body mass gain (mean ± standard error) in the period was similar (*p* = 0.328; Student's *t* test) between the control (145.3 ± 5.93%) and DBOI (154.1 ± 5.91%). However, the animals in both groups showed transient clinical signs such as piloerection and dyspnea. The clinical signs (Fig. 3) were more evident at the beginning of the exposure (between 30 and 240 min) and in the first two weeks, but only the animals exposed to DBOI had transient depression (evidenced by drowsiness and decreased response to stimuli) that lasted until the 4^th^ week.

Analyzing the behaviors in the open field test, the animals treated with DBOI were not different from the control in any of the analyzed characteristics (latency to move from the first quadrant (*p* = 0.765; Test *T* of independent samples), external crossing (*p* = 0.299; Test *T* of independent samples), internal crossings (*p* = 0.569; Test *T* of independent samples), rearing (*p* = 0.370; Test *T* of independent samples), grooming (*p* = 0.145; Test *T* of independent samples). These results explain that the spontaneous locomotor activity was normal and that the treatment caused no harm to the animals.

**Figure 4:**
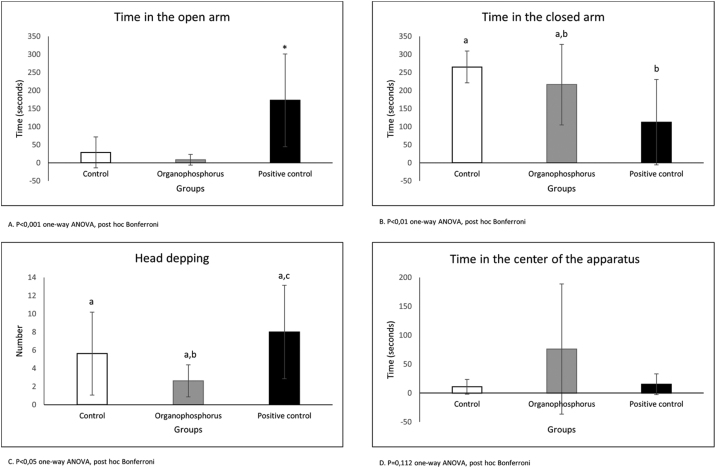
Plus maze test.

The plus maze test (Fig. 4) shows that animals treated with positive control (Diazepam) spent more time in the open arm (*p* < 0.001 one-way ANOVA, post hoc Bonferroni). The animals treated with positive control stayed less time in the closed arm, but the animals treated with DBOI were not different from positive control (*p* < 0.01 oneway ANOVA, post hoc Bonferroni). The time spent in the center of the apparatus shows no difference between the groups (*p* = 0.112 one-way ANOVA, post hoc Bonferroni). The head dipping demonstrates again the anxiolytic action of positive control (*p* < 0.05 one-way ANOVA, post hoc Bonferroni) and no difference between DBOI and positive control (*p* > 0.05 one-way ANOVA, post hoc Bonferroni).

Plasma cholinesterase values were similar (mean ± standard error: control = 5958.0 ± 145.0 U/L; DBOI = 5886.0 ± 112.9 U/L, respectively, *p* = 0.735; Student's *t* test). No macroscopic alterations were found in the organs of the animals used in the experimental protocol.

## Discussion

The present study has demonstrated ototoxicity induced by subchronic inhalation exposure of OP DBOI in rats, which was comparable to cisplatin-induced cochlear damage. The ototoxicity assessment protocol used in this study was consistent, considering all control groups (positive and negative) demonstrated expected behaviors. The choice of the rats as the experimental model for the DPOAE test to protocol is consistent with previous literature data, in which the authors concluded that DPOAE was the most frequent auditory evaluation used in the studies analyzed utilizing animal models, such as rats.^24^

The use of an animal model in the present study has allowed the identification of the outcome investigated (ototoxicity resulting from inhalation exposure to pesticide), controlling for possible bias (concomitant exposure to noise). Moreover, the inhalation route in an acoustically treated chamber, with an alternating exposure frequency that mimics the working day (5:2 days) has allowed us to consider that the effects observed here in rats could be comparable to those reported in the previous literature involving farmers.^3^, ^4^, ^6^

In addition, frequently previous studies evaluating cochlear function by otoacoustic emissions have used anesthetics, which have been reported as likely to cause changes in DPOAE responses.^25^, ^26^, ^27^ In the present research, it was possible to carry out the evaluations without anesthesia, due to the previous acclimatization of the animals to the instruments used in the auditory evaluations. Furthermore, the finding of ototoxicity secondary after the exposure to DBOI of rats corroborates previous studies that evaluated the hearing of individuals who had contact with pesticides.^6^, ^28^ Although studies in humans may present some bias, due to the difficulty to completely isolating the noise and other confounding factors inherent in the clinical history, such as exposure to vibration, occurrence of congenital pathologies such as toxoplasmosis, herpes and rubella, or even presbycusis, since damage to hearing may be multifactorial.^7^

Post-exposure results have indicated that even though dichlorvos has been an irreversible cholinesterase inhibitor, the concentration of the enzyme in the experimental group were similar to those in the control group. This fact suggests that levels of exposure to DBOI that do not cause significant enzymatic inhibition have ototoxic effect. Although studies have suggested the association between auditory damage and exposure to pesticides,^1^, ^2^, ^5^, ^6^, ^15^, ^16^ the current regulatory norm in Brazil^29^ (NR7) do not specify reference and sequential auditory exams for workers exposed to chemical agents, and the exposition to pesticides is evaluated by tests such as cholinesterase level. However, our results indicate that – even without alteration in cholinesterase levels – there was ototoxicity due to exposure to DBOI.

Concerning the effects of inhaled subchronic treatment DBOI in the Open Field and Plus Maze behaviors tests, in this research we demonstrate that DBOI administrated in Wistar rats was not an anxiogenic drug and had no impact on the locomotor activity. However, using another route of administration (oral), Silva et al. (2017) had different results in their study,^30^ in which Wistar rats were treated with chlorpyrifos (0.01, 0.1, 1 and 10 mg/kg/day) on gestational days (14–20). Male offspring behavior was evaluated on post-natal days 21 and 70 by the elevated plus-maze test and open field test. The results demonstrated that exposure to 0.1, 1 or 10 mg/kg/day of chlorpyrifos could induce anxiogenic behavior, but this signal was reversed on post-natal day 70.

The few clinical signs manifested by animals have indicated that toxicity to cochlear function, after the exposure to DBOI, was present at a level where rats did not exhibit relevant systemic toxicity – as in central nervous system (based on behavior tests) – only transient toxicity. The clinical signs were more frequent in the first hours of exposure, as well as in the first weeks of exposure, with no appearance in the last two weeks, which suggests a possible progressive tolerance to the same concentration of the substance. In addition, the transient alterations were reinforced by similar results between the groups regarding weight development.

The statistically significant difference between the control group and the positive control for injury was expected, since the ototoxicity of cisplatin has been well established in the literature.^19^, ^31^, ^32^, ^33^, ^34^, ^35^, ^36^ Additionally, the reduction of the DPOAE amplitude between the experimental group and the control group has indicated that exposure to OP actually could damage the cochlear function of the animals. The change in cochlear function values before and after DBOI exposure corroborates previous literature, which verified alterations in the cochlear morphology of guinea pigs due to exposure to an OP (0.3 mg/kg/day or 3 mg/kg/day during seven consecutive days, intraperitoneally), with alteration in the outer hair cells cytoarchitecture.^16^ This hypothesis is reinforced by the previous literature,^37^, ^38^ which indicated that exposure to insecticides could interact at the cochlear level.

Although the DPOAE test does not evaluate the hearing loss itself, the outer hair cells activity could be referred here. We highlight that the bilaterality of the 8 and 10 kHz damage in the experimental group is one of the characteristics of the sensorineural hearing loss secondary from exposure to ototoxic substances. In addition, the affected frequencies in animals exposed to DBOI are considered high, which also resembles the toxic agent induced hearing loss. On the other hand, the occurrence of loss in the high frequencies of 8 and 10 kHz bilaterally, but not in 12 kHz, is intriguing, and no similar result was found in the previous literature. This may be explained by the fact that usually DPOAE studies^1^, ^39^ do not consider 12 kHz frequency, and this might occur due to limitation of the equipment used.

The mechanism of hearing loss after exposure to organophosphates has not yet been fully elucidated.^2^ Ototoxicity secondary to some substances, such as gentamicin and cisplatin, has been related to possible alterations in the antioxidant functions in the sensory cells of the organ of Corti, especially the outer hair cells, due to lipid peroxidation.^40^

This study has potential limitations. Although Auditory Brainstem Response (ABR) testing would be a better indicator of cochlear function and the presence of hearing impairment than DPOAE, the ABR test was not performed, since the ABR equipment was in a building inaccessible to animals. DPOAE could be performed, since the equipment is portable, and can be taken where the animals were housed. In addition, the institution's equipment did not have the ABR screening function (ABR-A). The second limitation is the lack of previous research studies analyzing frequencies such as 10 kHz and 12 kHz, which makes it difficult to compare our results with another findings (such as statistical difference at 8 kHz and 10 kHz in the DBOI group, but not at 12 kHz). Another limitation is that the evaluation of DPOAE without the measurement of antioxidant enzymes in the auditory system does not justify the statement that ototoxicity to DBOI could be caused by dysfunction in antioxidant enzymes; however, it suggests that this may be related to it, as the decrease or absence of DPOAE can indicate damage in hair cells function. In order to fully clarify this mechanism, new functional research studies will be necessary, associated with methods that evaluate antioxidant enzymes and the morphology of hair cells, such as scanning electron microscopy or even fluorescence microscopy.

## Conclusion

DBOI caused ototoxicity in the animals evaluated, comparable to cisplatin-induced damage in DPOAE, without significant systemic effects, with loss at frequencies of 8 and 10 kHz bilaterally.

## Funding

This study was financed in part by the Coordenação de Aperfeiçoamento de Pessoal de Nível Superior – Brasil (10.13039/501100002322CAPES) – Finance Code 001.

## Conflicts of interest

The authors declare no conflicts of interest.
